# TIGIT agonism as a therapeutic strategy to suppress inflammation in hidradenitis suppurativa

**DOI:** 10.3389/fimmu.2026.1761782

**Published:** 2026-04-01

**Authors:** Michal Kidacki, Anjali Jaiswal, Christina Cho, Samantha Prince, Rachel Breidbart, Henry Hsia, Matthew D. Vesely, Lieping Chen

**Affiliations:** 1 Department of Dermatology, Yale School of Medicine, New Haven, CT, United States; 2 Department of Immunobiology, Yale School of Medicine, New Haven, CT, United States; 3 Department of Surgery (Plastic and Reconstructive Surgery), Yale School of Medicine, New Haven, CT, United States; 4 Department of Internal Medicine (Oncology), Yale School of Medicine, New Haven, CT, United States

**Keywords:** hidradenitis suppurativa, HS, immune checkpoint, inflammation, PVRL3, TIGIT

## Abstract

Hidradenitis suppurativa (HS) is a chronic, heterogeneous inflammatory skin disorder with limited therapeutic options. The immune checkpoint receptor TIGIT is emerging as a regulator of chronic inflammation, yet its role in HS remains unknown. Here, we investigated TIGIT and its ligands in HS using tissue profiling, transcriptomics, and an ex vivo functional explant model. A tissue microarray containing 52 HS, 9 ruptured follicular cysts, and 4 normal skin samples demonstrated significantly increased TIGIT expression in HS lesions. In contrast, the TIGIT ligand PVRL3 was significantly decreased in HS, a finding also observed in a publicly available RNA-seq dataset. Because TIGIT suppresses inflammation only when adequately engaged by its ligands, reduced PVRL3 may impair inhibitory checkpoint signaling in HS. To test whether TIGIT engagement could suppress HS inflammation, we developed an ex vivo explant model using freshly obtained HS lesional tissue. The system was validated using triamcinolone, a corticosteroid, which consistently reduced IL-6 production. Treatment with a TIGIT agonist antibody significantly reduced IL-6 by 72 hours. These findings provide the first functional evidence that TIGIT activation attenuates inflammatory pathways in HS, supporting TIGIT agonism as a potential therapeutic strategy.

## 1 Introduction

Hidradenitis suppurativa (HS) is a chronic, recurrent inflammatory skin disease characterized by painful nodules, abscesses, and draining sinus tracts that lead to scarring and substantial morbidity (1). HS etiology remains incompletely understood – involvement of follicular immune activation, persistent innate–adaptive crosstalk, and inflammatory pathways driven by cytokines such as TNF, IL-1β, and IL-17/IL-23, alongside neutrophil-enriched inflammation and extracellular matrix remodeling have been proposed (2). The limited efficacy of current therapies highlights the need to identify mechanistically informed new pathways driving persistent inflammation (2–5).

Inhibitory immune checkpoints are critical physiologic mechanisms that constrain immune-mediated tissue injury. Perturbation of checkpoint signaling can contribute to chronic inflammatory disease, and checkpoint blockade has been reported to exacerbate HS (3, 6, 7) (8, 9). TIGIT (T-cell immunoreceptor with Ig and ITIM domains) is an inhibitory immune checkpoint expressed on activated T cells and NK cells, which are found in HS lesions (10, 11). Canonically, TIGIT can dampen inflammatory responses, but its inhibitory function depends on ligand engagement, primarily through PVR (CD155) and PVRL3 (CD113), which can compete with the activating receptor CD226 (DNAM-1) for binding (12). Perturbations in ligand expression or receptor–ligand balance may impair checkpoint activity, potentially favoring persistent inflammation. Although TIGIT has been examined in autoimmune disease and cancer, the effects of TIGIT agonism in HS have not been explored in HS despite TIGIT signaling directly interfacing with pathways central to HS immunopathology (13–16) TIGIT engagement suppresses T cell–derived cytokines including TNF, IL-17, and IFN-γ, modulates antigen-presenting cell activation, and can restrain CD226-mediated costimulatory signaling that promotes Th17 polarization and effector responses (17–19) Dysregulated TIGIT–CD226 balance has been associated with heightened IL-17 and TNF production in chronic inflammatory settings (19). Given that HS lesions are characterized by TNF- and IL-1β–driven inflammation, Th17 skewing, neutrophil recruitment, and persistent follicular immune activation, impaired TIGIT agonism could plausibly contribute to sustained cytokine production and neutrophil-enriched inflammation within the HS microenvironment. We hypothesize that TIGIT is under-agonized in HS and engagement of this immune checkpoint may lead to decrease in inflammation.

In this study, we characterize TIGIT and its ligands in HS using tissue microarray analysis, transcriptomic validation, and a functional ex vivo explant HS model that enables mechanistic testing directly in human HS tissue. We report ligand imbalance, TIGIT^+^ cell enrichment, and demonstrate that agonism of TIGIT suppresses key inflammatory mediators produced by HS lesions. These findings provide preliminary evidence of TIGIT as an active pathway with therapeutic potential in HS.

## 2 Methods

### 2.1 Tissue microarray and immunostaining

All immunostaining was performed under standardized conditions using optimized protocols established prior to analysis of HS samples. Antibodies used in this study were first optimized on a multi-organ tissue microarray containing normal human liver, lung, heart, brain, lymph node, placenta, ovary, and testis. Observed staining patterns were compared against publicly available reference data from the Human Protein Atlas to confirm expected cellular localization and exclude nonspecific signal. Only antibodies demonstrating concordant tissue distribution and expected subcellular localization were advanced to HS tissue analysis. A tissue microarray (TMA) was constructed containing 64 cores: HS lesional skin (n = 52), ruptured follicular cysts (n = 9), and normal skin (n = 4). HS samples spanned Hurley stages I–III and included deep dermal or perisinusoidal sampling regions. Formalin-fixed, paraffin-embedded HS tissue sections were deparaffinized (60 °C overnight, xylene and graded ethanol battery) and subjected to heat-mediated antigen retrieval in 1 mM EDTA (pH 9) at 96 °C. After blocking endogenous peroxidase (Dako Dual Enzyme Block) and incubation in 5% horse serum, slides were incubated with primary antibodies (PVR, M00664-2, BosterBio; TIGIT, BLR047F,Biocare Medical). Secondary antibodies were applied sequentially, tyramide amplified and quenched with benzoic hydrazide. Nuclei were counterstained with DAPI, and slides were mounted in ProLong Diamond. For PVRL3 immunohistochemistry slides were deparaffinized as above and subjected to citrate antigen retrieval (pH 6.0, 96 °C). Endogenous peroxidase was blocked (Dako Dual Enzyme Block), and slides were incubated in 5% horse serum before application of the primary antibody (PVRL3, EPR26326-22, Abcam). After TBST washes, HRP-conjugated anti-rabbit polymer (PolyVision) and DAB-nickel chromogen (Vector SK-4100) were used for detection. Sections were counterstained with hematoxylin, differentiated, blued, dehydrated, and mounted in VectaMount. For CD8/TIGIT immunofluorescence staining – whole tissue sections of n=5 ruptured follicular cyst (RFC) and n=5 HS samples were used. Protocol as above. CD8 antibody used CF802079 from Origene. Images were processed in QuPath, where cells were segmented using the built-in cell detection algorithm and PVR, TIGIT, or PVRL3 positivity was calculated as the proportion of all detected cells. All HS, RFC, and normal skin cores had the same linear detection range to ensure comparability of signal intensity across samples. No cores were excluded as outliers. Expected cellular localization patterns were observed in all groups. Image acquisition and analysis parameters were held constant across samples to minimize batch or thresholding bias.

### 2.2 Transcriptomic dataset analysis

Public RNA-seq data (GSE175990) comparing HS and healthy skin were analyzed using a pseudobulk approach with a Wilcoxon rank-sum test. P-values were adjusted using Benjamini–Hochberg false discovery rate.

### 2.3 Ex vivo HS explant culture

Fresh HS lesional tissue was obtained under Yale IRB approval (#2000035999) and processed within 2 hours of excision. For each patient, comparable full-thickness 4-mm punch biopsies obtained from inflamed portions of skin were distributed across experimental conditions. Only explants of similar size and depth were used within each experiment to reduce variability in tissue mass. Explants were cultured in DMEM supplemented with 0.1% FBS, penicillin–streptomycin (Gibco), and amphotericin B (Cytiva, 2.5µg/ml)and maintained at 37 °C, and conditioned media were collected every 24 hours, spun down at 3000g to remove cell debris and frozen in -80C for later analysis. Triamcinolone acetonide was added at 0.4 µg/mL for the Kenalog-treated group (n = 9). For TIGIT agonism (a15153G, Biolegend) experiments, explants from HS patients (n = 8) were treated with the 0.3 µg/mL concentration of the antibody. Explant viability was assessed histologically. Representative explants were fixed at the conclusion of culture and evaluated by hematoxylin and eosin staining.

### 2.4 Cytokine quantification

Cytokines from collected conditioned media were measured in duplicate using ELISA for IL-6 (LS-F31403-1, LS Bio) with matched assays for IL-1β (DY201-05, R&D Systems) and MMP9 (DMP900, R&D Systems) according to manufacturers protocols. Cytokine concentrations were analyzed as absolute values per explant without normalization to tissue weight. The paired within-patient design was used to mitigate inter-lesional variability.

### 2.5 Statistics

Sample sets were tested for normality. In the small sample size sets and non-normal distribution we performed nonparametric bootstrap resampling (10,000 iterations) to estimate mean differences for group comparisons in immunofluorescence datasets. If samples passed normality testing we performed parametric student t test. RNA-seq analyses used Wilcoxon rank-sum test with Benjamini-Hochberg false discovery rate. Cytokine correlations were assessed using Pearson correlation and linear regression. Significance was defined as p < 0.05.

## 3 Results

### 3.1 TIGIT is increased in HS lesional tissue

**Figure 1:**
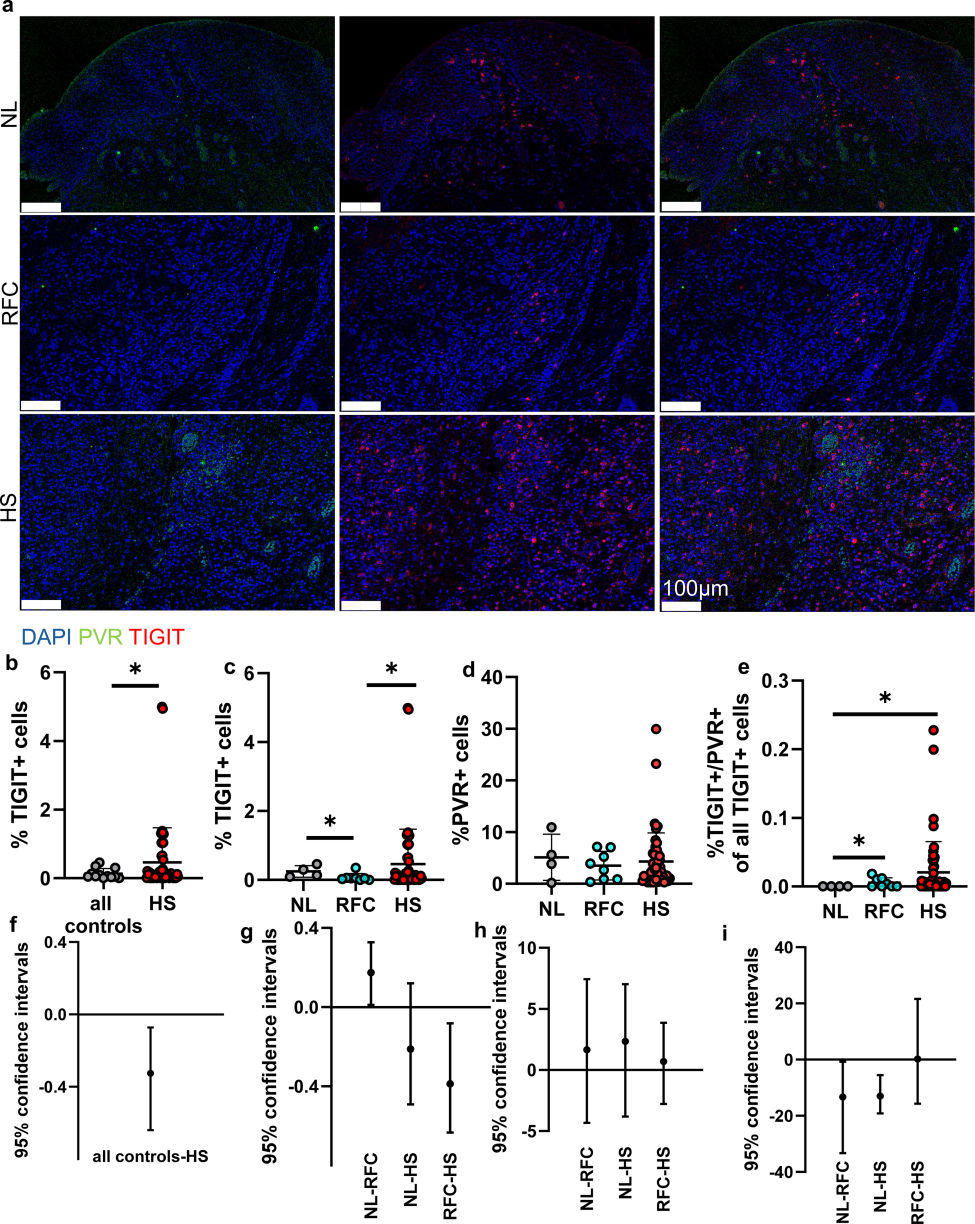
**(A)** Immunofluorescence images showing PVR (green), TIGIT (red), and DAPI (blue) in normal control skin(NL), hidradenitis suppurativa (HS), and follicular cyst (RFC) samples. **(B–E)**Quantification of TIGIT*, PVR* and PVR/TIGIT* as percent of all TIGIT+ cells was performed in QuPath, normalized to all detected cells. **(F–I)** Forest plots showing bootstrap estimates of between-group mean differences with 95% confidence intervals for the corresponding quantifications panels above **(B–E)**; the vertical line denotes no difference (0). Sample sizes: control (n = 4), HS (n = 52), FC (n = 8). *p<0.05. Scale bar = 100 μm. Bars represents means.

To investigate TIGIT expression in HS, we constructed a tissue microarray that included representative lesional samples across Hurley stages I through III, together with detailed annotation of patient gender and the anatomic depth of each selected section, as summarized in **Table 1**. HS cores were chosen to capture the range of inflammatory architectural patterns encountered in clinical disease, including both deep dermal and perisinusoidal regions. As comparative controls, we included samples from ruptured follicular cysts (RFC) and normal skin. RFC were selected because they produce acute follicular disruption with associated inflammation, and histologically represent the closest related entity to HS, yet they follow a very different clinical course that resolves rather than progressing to chronic draining tunnels or recurrent inflammation. Immunofluorescence analysis of the TMA demonstrated a significant increase in TIGIT positive cells in HS lesions compared with all controls expressed as percent positive cells out of all cells identified in the cores (**Figures 1A, B, F**). When controls were divided into normal skin and RFC, HS compared to only RFC (but not to normal skin) remained significantly elevated (**Figures 1C, G**). Quantitatively, TIGIT expression was approximately 2.5 fold higher in HS relative to normal and 5 fold higher than RFC (**Figures 1C, G**). In contrast to TIGIT, PVR expression did not differ between HS, RFC, and normal skin samples (**Figures 1D, H**). The amount of PVR positive/TIGIT positive double expressing cells as a proportion to all TIGIT positive cells was significantly increased only compared to normal skin but there was no difference between HS and its inflammatory control, RFC (**Figures 1E, I**). These findings indicate that TIGIT upregulation in HS occurs independently of proportional increases in its ligand PVR and support the possibility that TIGIT elevation reflects chronic unagonized immune activation. There was no statistical significance in PVR or TIGIT expression between Hurley stages and gender within the sample set (data not shown).

**Table 1:** Characteristics of patient samples used in the tissue microarray.

Hurley stage	HS (n = 52)	Ruptured follicular cysts (n = 9)	Normal skin (n = 4)
I	4	–	–
II	16	–	–
III	23	–	–
Unknown	9	–	–
Gender			
Female	32	6	4
Male	20	3	0
Core depth			
Deep dermis	35	–	–
Perisinus	15	–	–
Superficial	2	–	–

### 3.2 The TIGIT ligand PVRL3 is selectively reduced in HS

**Figure 2:**
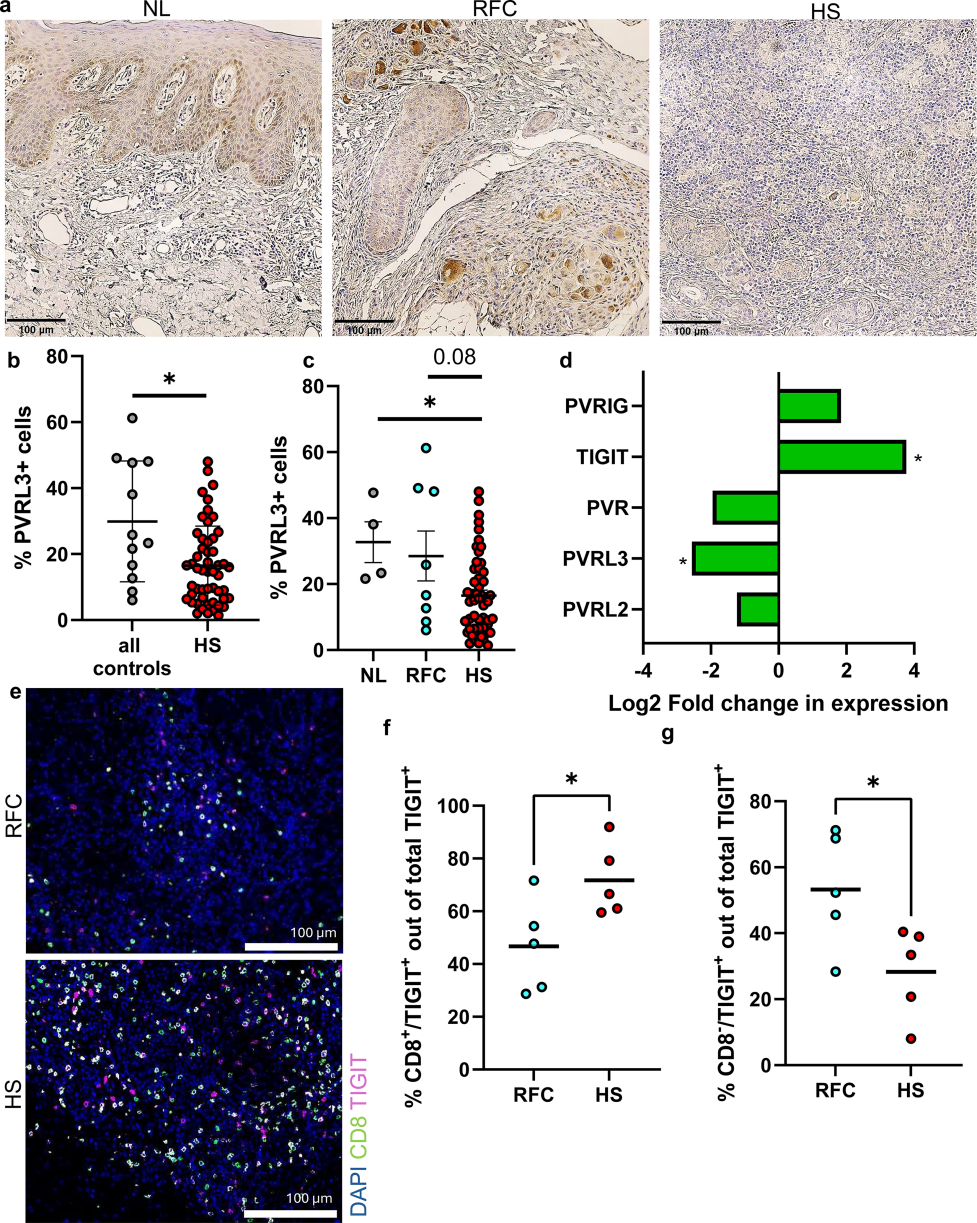
**(A)** Immunohistochemistry images showing PVRL3 staining in normal control skin (NL), hidradenitis suppurativa (HS), and follicular cyst (RFC) samples. **(B, C)** Quantification of PVRL3 positive cells was performed in QuPath and expressed as the Opercentage of all detected cells. Sample sizes: control (n = 4), HS (n = 52), FC (n = 8). Statistical analysis via nonparametric bootstrap inference. Scale bar = 100 μm. **(D)** Analysis of publicly available dataset - Log2 fold change comparing HS vs healthy skin - TIGIT and its ligands (GSE175990). Statistical analysis was performed using Wilcoxon rank- sum test with Benjamini-Hochberg false discovery rate. **(E)** Representative immunofluorescence images of CD8 and TIGIT co-staining in HS and RFC samples. **(F)** Percentage of CD8+TIGIT+ cells expressed as a proportion of all TIGIT+ cells. **(G)** Percentage of CD8-TIGIT cells expressed as a proportion of all TIGIT+ cells. CD8 and TIGIT subset analysis was performed in a sample set of HS (n = 5) and RFC (n = 5). Statistical analysis was performed using a nonparametric unpaired t test. Bars represent means. *p<0.05.

Given that TIGIT requires ligand engagement to exert inhibitory signaling, we next evaluated PVRL3, a ligand with lower affinity than PVR (10). Immunohistochemistry showed that PVRL3^+^ cells were reduced by roughly two-fold in HS compared with controls (**Figures 2A, B**), and PVRL3^+^ cell frequency was lower in HS than in both RFC ([p<0.08) and normal skin (p<0.05) (**Figure 2C**). Independent transcriptomic analysis of the GSE175990 dataset confirmed significant PVRL3 transcript reduction in HS compared with healthy skin (**Figure 2D**), mirroring protein-level findings and supporting selective ligand insufficiency rather than global alteration of all TIGIT ligands (20). Together, these results suggest a receptor–ligand imbalance wherein TIGIT is elevated while PVRL3 is diminished.

### 3.3 TIGIT positive populations shift in HS as compared to RFC

To explore whether TIGIT upregulation in HS was associated with specific lymphocyte subsets, we performed CD8 and TIGIT co staining in a subset of samples (n = 5 HS and n = 5 RFC). Representative images are shown in **Figure 2E**. When expressed as a proportion of all TIGIT positive cells, the percentage of CD8^+^TIGIT^+^ cells was significantly higher in HS compared with controls (**Figure 2F**), whereas the percentage of CD8^-^TIGIT^+^ cells was correspondingly lower (**Figure 2G**). These proportional differences suggest a shift in the relative composition of TIGIT expressing lymphocyte subsets in HS. Given the small cohort size, these findings will require validation in a larger, more powered cohort. However, the absolute percentages of CD8^+^TIGIT^+^ or CD8^-^TIGIT^+^ cells per sample were not significantly different between groups, which indicates that the primary change is in the distribution of TIGIT expressing populations rather than in their absolute abundance (data not shown).

### 3.4 TIGIT agonism suppresses IL-6 in HS explants

**Figure 3:**
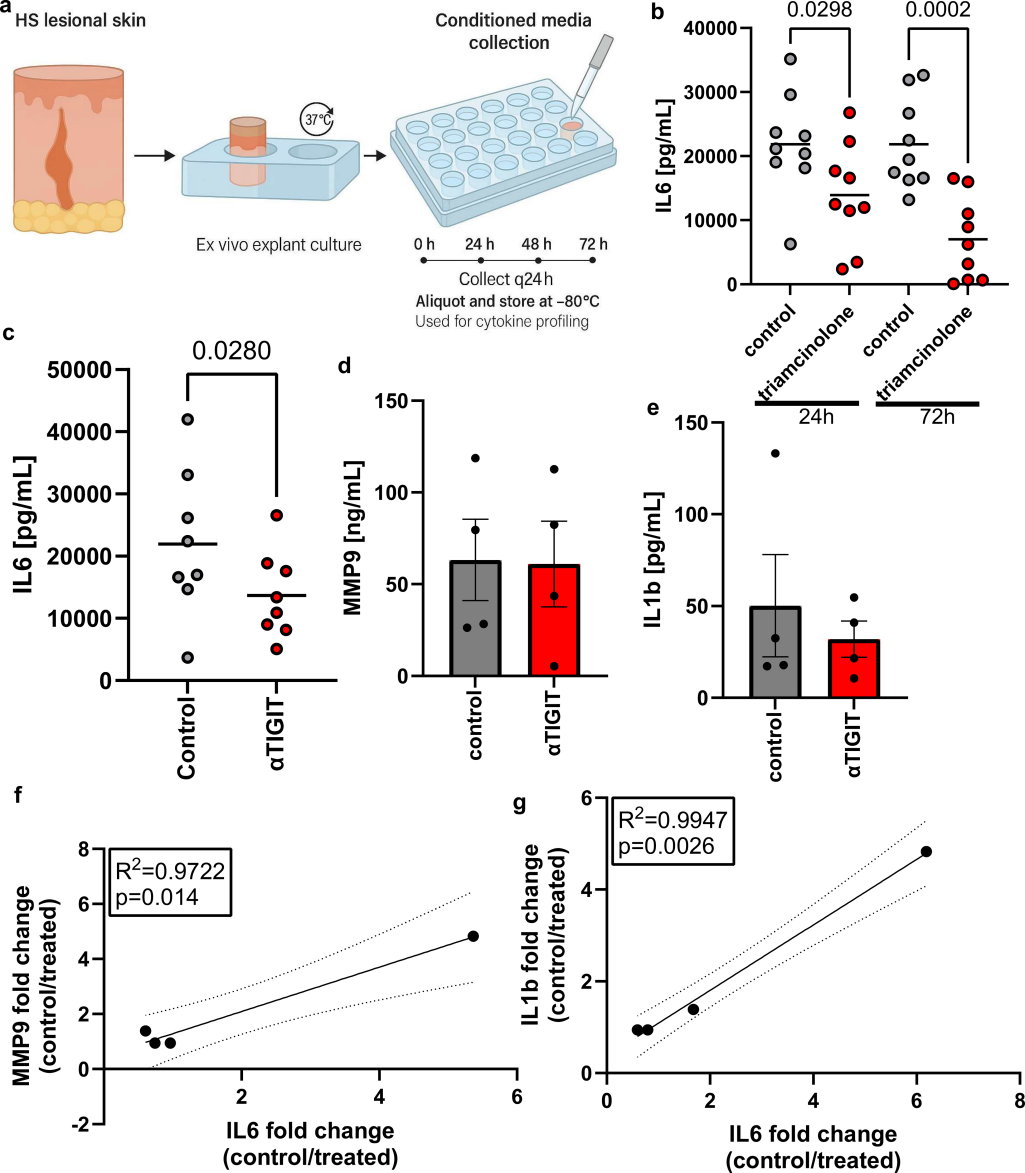
**(A)** Schematic of the ex vivo explant workflow. Full-thickness punch biopsies from HS lesions were placed upright in individual wells containing culture medium, incubated at 37 °C, and conditioned media were collected every 24 h for cytokine profiling. **(B)** Proof-of-concept experiment demonstrating that Kenalog (0.4 μg/mL) reduces IL-6 secretion from HS explants at 24 h and 72 h **(C)** Treatment of HS explants with anti- TIGIT antibody resulted in a significant reduction in IL-6 by 72 h Bars represent means. **(D)** MMP9 and **(E)** IL1b concentrations measured in conditioned media from HS explants. **(F, G)** Correlation analyses showing that IL-6 fold-change strongly correlates with reductions in MMP9 **(F)** and IL-1β **(G)**. Statistical analyses were performed using paired student's t-test.

The HS field has identified many inflammatory pathways through transcriptional and histologic profiling, but very few of these pathways have been functionally tested in intact human tissue, and almost none have been validated through direct perturbation studies (21). To establish a system capable of mechanistic testing, we developed an ex vivo HS explant culture model and first assessed its cytokine output profile (**Figure 3A**). Among cytokines consistently detectable across samples, IL6 was selected as the primary readout because it is the only soluble mediator repeatedly shown to correlate with disease severity and with clinical treatment response, whereas other cytokines in HS have shown inconsistent or patient specific variation (22–25). As an internal positive control, we treated explants with triamcinolone acetonide at 0.4 µg/mL. Triamcinolone led to a two and a three fold reductions in IL6 secretion at 24 hours and at 72 hours, respectively (**Figure 3B**). This reproducible suppression across nine independent patient samples demonstrates that the model reliably detects modulation of inflammation. We next evaluated whether TIGIT activation could modulate inflammatory output. Explants were treated with an agonistic TIGIT antibody (a15153G, 0.3 µg/mL) (26, 27). TIGIT agonism produced a modest but significant decrease in IL6 at 72 hours with an average of 1.6 fold reduction compared with paired controls across eight independent patient samples (**Figure 3C**). Because TIGIT agonism targets a distinct regulatory pathway, a smaller magnitude of IL6 suppression was expected compared with corticosteroids, which act broadly across multiple inflammatory axes. In direct comparisons of absolute concentrations, neither MMP9 nor IL-1β demonstrated statistically significant differences between treatment conditions, reflecting inter-explant variability in baseline inflammatory output (**Figures 3D, E**). However, when changes were analyzed relative to IL-6 modulation within each sample, a coordinated pattern emerged. IL-6 fold-change strongly correlated with fold-change in MMP9 (R² = 0.97, p = 0.014) and IL-1β (R² = 0.99, p = 0.0026) (**Figures 3F, G**). These findings suggest that TIGIT engagement does not selectively modulate IL-6 alone but instead produces coupled attenuation of interconnected inflammatory pathways.

## 4 Discussion

This study identifies TIGIT as a functionally relevant inhibitory pathway in HS and demonstrates that TIGIT agonism suppresses inflammatory cytokine production in lesional human tissue. No prior work has investigated the role of TIGIT in HS etiology. The finding that TIGIT is significantly elevated in HS aligns with this broader pattern of checkpoint upregulation in chronic inflammatory skin disease (7).

A key observation in this study is the discordance between TIGIT expression and the abundance of its ligands. PVR was unchanged across groups, but PVRL3 was significantly decreased at both the protein level and in independent transcriptomic data. TIGIT requires ligand engagement to mediate its inhibitory effects, and ligand insufficiency can limit TIGIT function and sustain inflammation, a concept described in chronic autoimmune settings (12–16). Our findings suggest that a similar receptor ligand imbalance may exist in HS, where TIGIT is abundant but inadequately engaged because of reduced PVRL3. An important consideration is that TIGIT binds multiple ligands with differing affinities. PVR binds TIGIT with higher affinity than PVRL3, and therefore one might question whether reduced PVRL3 expression is functionally meaningful when PVR levels are preserved. However, checkpoint signaling is determined not solely by ligand affinity but by the combined effects of ligand abundance, cellular distribution, and competitive receptor engagement within the tissue microenvironment. In HS skin, we observed preserved PVR expression but a significant reduction in PVRL3 at both the protein and transcript level. Even modest shifts in total ligand availability may influence the balance between inhibition and stimulation.

An important limitation of this study is that normal skin controls were obtained from abdominoplasty and breast reduction procedures with unknown and likely prolonged warm ischemia times before fixation. Immune markers such as TIGIT can be sensitive to ischemic delay, and this variable may partly contribute to elevated TIGIT positive cell frequency in HS relative to normal controls. Because HS is characterized by dense inflammatory infiltrates and our primary biological question concerned checkpoint regulation within inflamed follicular tissue, RFC were used as the principal comparator. Normal skin, which contains relatively sparse immune cell populations, was included primarily as a baseline reference rather than the central inferential control group. Importantly, TIGIT expression remained significantly elevated in HS when compared with RFC controls, which represent smaller surgical specimens with shorter excision-to-fixation times and acute inflammatory follicular pathology. This comparison supports the biological validity of TIGIT upregulation in HS independent of ischemia-related artifact or differences in baseline immune cell density.

Using an ex vivo explant system, we demonstrated that TIGIT agonism suppresses HS associated inflammation. Triamcinolone acetonide produced a broad reduction in IL6, which confirmed model responsiveness and established a clinically relevant benchmark. TIGIT agonism achieved a 1.6 fold IL6 reduction by 72 hours, which is modest but biologically meaningful. Only a single TIGIT agonist concentration was tested, which leaves open the possibility that the effect is dose dependent, patient dependent, or requires longer exposure to reach maximal inhibition. Moreover, our study was performed in a small cohort and evaluated only a single agonist concentration; therefore, future studies will be required to define optimal dosing parameters and to identify the patient population most likely to benefit from TIGIT agonist–based therapy. Importantly, checkpoint targeted strategies have the advantage of narrower, more pathway specific immunomodulation compared with corticosteroids, which may reduce long term adverse effects associated with broad immunosuppression. Histologic evaluation of representative explants demonstrated preserved tissue architecture and viable cellularity over the culture period in the majority of samples, although some degree of inflammatory cell attrition was observed, consistent with expected ex vivo tissue dynamics. Because each patient served as their own internal control and comparable punch biopsies were distributed across treatment conditions, relative fold-change comparisons remain interpretable despite inherent variability in cellular density. Future studies incorporating dose-response analyses and quantitative normalization to tissue mass will further refine these observations. The observed correlation of MMP9 and IL-1β with IL6 reduction, suggests coordinated down modulation of multiple inflammatory axes that are central to HS pathogenesis (28, 29). These analyses were performed on a subset of conditioned media that had sufficient residual volume, so they should be interpreted as preliminary. Larger explant cohorts and broader cytokine profiling will be necessary to determine the full inflammatory signature downstream of TIGIT engagement. In all functional assays, explants with overt contamination or tissue dissolution were excluded to maintain biological interpretability.

Taken together, these findings integrate tissue level profiling, transcriptomic analysis, and functional perturbation to provide the first direct evidence that TIGIT activation attenuates inflammatory pathways in HS. These results add to a growing body of literature that implicates checkpoint dysregulation in inflammatory skin conditions (6–9) and highlight TIGIT agonism as a promising therapeutic strategy. Future studies that include dose response testing, ligand restoration, and *in vivo* modeling will be essential to define the therapeutic potential and optimal clinical application of TIGIT directed interventions in HS.

## Data Availability

The raw data supporting the conclusions of this article will be made available by the authors, without undue reservation.
